# Clonal Structure of Rapid-Onset MDV-Driven CD4+ Lymphomas and Responding CD8+ T Cells

**DOI:** 10.1371/journal.ppat.1001337

**Published:** 2011-05-05

**Authors:** William N. Mwangi, Lorraine P. Smith, Susan J. Baigent, Richard K. Beal, Venugopal Nair, Adrian L. Smith

**Affiliations:** 1 Avian Infectious Disease Programme, Institute for Animal Health, Compton, Berkshire, United Kingdom; 2 Department of Zoology, University of Oxford, Oxford, United Kingdom; Freie Universitaet Berlin, Germany

## Abstract

Lymphoid oncogenesis is a life threatening complication associated with a number of persistent viral infections (e.g. EBV and HTLV-1 in humans). With many of these infections it is difficult to study their natural history and the dynamics of tumor formation. Marek's Disease Virus (MDV) is a prevalent α-herpesvirus of poultry, inducing CD4+ TCRαβ+ T cell tumors in susceptible hosts. The high penetrance and temporal predictability of tumor induction raises issues related to the clonal structure of these lymphomas. Similarly, the clonality of responding CD8 T cells that infiltrate the tumor sites is unknown. Using TCRβ repertoire analysis tools, we demonstrated that MDV driven CD4+ T cell tumors were dominated by one to three large clones within an oligoclonal framework of smaller clones of CD4+ T cells. Individual birds had multiple tumor sites, some the result of metastasis (i.e. shared dominant clones) and others derived from distinct clones of transformed cells. The smaller oligoclonal CD4+ cells may represent an anti-tumor response, although on one occasion a low frequency clone was transformed and expanded after culture. Metastatic tumor clones were detected in the blood early during infection and dominated the circulating T cell repertoire, leading to MDV associated immune suppression. We also demonstrated that the tumor-infiltrating CD8+ T cell response was dominated by large oligoclonal expansions containing both “public” and “private” CDR3 sequences. The frequency of CD8+ T cell CDR3 sequences suggests initial stimulation during the early phases of infection. Collectively, our results indicate that MDV driven tumors are dominated by a highly restricted number of CD4+ clones. Moreover, the responding CD8+ T cell infiltrate is oligoclonal indicating recognition of a limited number of MDV antigens. These studies improve our understanding of the biology of MDV, an important poultry pathogen and a natural infection model of virus-induced tumor formation.

## Author Summary

Many viral infections target the immune system, making use of the long lived, highly proliferative lymphocytes to propagate and survive within the host. This characteristic has led to an association between some viruses such as Epstein Barr Virus (EBV), Human T cell Lymphotrophic Virus-1 (HTLV-1) and Mareks Disease Virus (MDV) and lymphoid tumors. We employed methods for identifying the T cell receptor repertoire as a molecular bar-code to study the biology of MDV-induced tumors and the anti-tumor response. Each individual contained a small number of large (high frequency) tumor clones alongside some smaller (lower frequency) clones in the CD4+ T cell population. The tumor infiltrating CD8+ T cell response was highly focused with a small number of large clones, with one representing a public CDR3 sequence. This data is consistent with the recognition of a small number of dominant antigens and understanding the relationship between these and protective immunity is important to improve development of new vaccination strategies. Collectively, our results provide insights into the clonal structure of MDV driven tumors and in the responding CD8+ T cell compartment. These studies advance our understanding of MDV biology, an important poultry disease and a natural infection model of virus-induced tumor formation.

## Introduction

Virus driven lymphoid oncogenesis is a serious consequence of infection with a wide range of herpes and retroviral pathogens in a variety of hosts. Major lymphoma-associated infections of humans include Epstein Barr virus (EBV) and Human T cell lymphotropic virus (HTLV) [1], [2]. With both EBV and HTLV tumor progression is a relatively rare event considering the prevalence of infection and the persistent nature of the virus [2], [3]. In contrast, Marek's Disease Virus (MDV) is a widespread, oncogenic α-herpesvirus infection of chickens which readily causes lymphoid tumors and has immense impact on the poultry industry [4]. The oncogenicity of MDV, combined with the ability to vaccinate against tumor formation make the MDV-chicken system an excellent natural infection model for understanding the biology and treatment of viral induced lymphomas [1], [5]–[7].

The spread of MDV occurs through the inhalation of infectious particles in dust. After a brief lytic phase in B lymphocytes (∼2 to 7 days post infection [dpi]), MDV establishes a life-long latent infection in CD4+ T lymphocytes [8]. The life-cycle is completed by transfer of the MDV to the feather follicle epithelium [8]. In susceptible birds, MDV infection leads to a high incidence of CD4+ T cell tumors (up to 100%) in a wide range of organs including heart, liver, ovary, testes, lungs and skin [9]–[14]. These CD4+ tumors express high levels of CD30, a tumor necrosis factor receptor II family member, also over-expressed on human lymphomas with diverse etiologies [5]. MDV latency and tumor formation is dependent upon viral encoded genes such as EcoRI-Q (meq), a c-Jun related molecule [15]–[17].

The penetrance (up to 100%) and temporal reproducibility of tumor appearance after infection (within 3 to 4 weeks) in susceptible lines of bird raises important questions regarding tumor clonality. These include the clonality of transformed cells in individual sites and between sites where multiple discrete solid tumors are evident in a single individual. The MDV genome readily integrates into the host cell genome particularly at telomeric or sub-telomeric locations [18], [19]. The profile of MDV integration within the tumor host cell suggested restricted clonality of most Marek's Disease-derived cell lines and cells taken from tumor sites [18], [19]. Between two and twelve independent integration sites were detected in each sample and the pattern of integration was stable over time in culture. In contrast, analysis of T cell receptor (TCR) Vβ family usage in CD30^hi^ cells from primary lymphomas led to the conclusion that MD tumors were polyclonal [10]. During the analysis of MDV integration patterns, samples obtained from a single chicken contained at least two major distinct patterns [19] suggesting at least two independent transformation events. These data coupled with the possibility of favoured sites for MDV integration [e.g. telomeric or sub-telomeric preference, [19]] suggest that a non-viral integration site dependent analysis of clonality would be appropriate. Since the tumors are derived from CD4+ T cells, the clonally expressed T cell receptor (TCR) would be an appropriate target for the molecular definition of tumor clonality.

The development of successful anti-tumor vaccines against MDV has been critical in poultry production and led to the proposal of utility for MDV as a model for developing vaccines against other lymphoma-inducing viral infections reviewed in [1], [6]. The vaccines are highly effective at preventing tumor formation but fail to eliminate infection or block transmission over prolonged periods [20]. Periodically, circulating strains of MDV develop enhanced pathogenicity and vaccine break has necessitated the development of different generations of vaccines over the past 50 years [Reviewed in [21], [22]]. The success of vaccination indicates acquisition of protective adaptive immunity and both antibody and T cell responses are readily detected [23], [24]. Other evidence for immune protection includes the association of genetic resistance with the MHC (B locus) haplotype [25]–[29]. Similarly, natural infection induces measurable natural killer cell, antibody, T cell and cytokine and interferon responses [30]–[34]. The highly cell associated nature of MDV supports the notion that cell mediated responses may predominate in protective immunity (reviewed by [23], [35], [36] with the CD8+ T cell mediated cytotoxic killing demonstrated in several studies [37]–[39]. The cytotoxic activity in MHC B^19^ and B^21^ homozygous chickens was focussed on the MDV-encoded pp38, meq and gB antigens [38]. Importantly, transient depletion of CD8+ T cells rendered chickens more susceptible to infection with MDV [40]. The response to persistent viral infections in humans is often characterised by cytotoxic T cells specific to latency-associated antigens. Indeed, large clones of T cells are readily detected during infection with CMV [41], [42] and EBV [2], [43], [44]. This type of clonal structure within CD8+ T cells is indicative of a response focussed on very few antigens.

The issue of tumor clonality and the nature of the CD8+ T cell response during MDV infection prompted application of the T cell receptor repertoire analysis tools we have recently developed for the chicken [45]. The chicken TCRβ locus in chickens is much simpler than in mammals containing 13 Variable (V), 1 Diversity (D), 4 Joining (J) segments and 1 C segment [45]–[49]. The Vβ segments group into two families, which simplifies global analysis of the chicken TCR repertoire. We applied a combination of CDR3 length analysis (spectratyping) and sequencing of the VDJ-junction (also known as the complementary determining region 3 [CDR3]) to define the clonality of MDV cell lines and different populations of cells from tumors or other sites within MDV infected birds. These approaches revealed clonal structure within MDV tumors (but not always monoclonal) and a pattern of shared and distinct clonal origin in different sites within a single individual. Analysis of the tumor infiltrating and splenic CD8+ T cells allowed identification of large T cell clones within an oligoclonal framework of responding CD8+ T cells.

## Materials and Methods

### Experimental infection

Inbred line P (MHC, B^19/19^) white leghorn chickens were reared pathogen free at the Poultry Production Unit of the Institute for Animal Health. One-day-old birds were infected with of MDV strain RB-1B [50] by intra-abdominal injection of ∼1000 pfu cell associated virus and observed for the development of MD using methods described previously [51], [52]. Two of the birds (15 and 16) were sentinel birds and infected by exposure to experimentally infected birds. Birds were reared with *ad libitum* access to water and vegetable-based diet (Special Diet services, Witham, UK) and wing-banded to allow identification of individuals.

### Ethics statement

This study was carried out according to the guidance and regulations of the UK Home Office with appropriate personal and project licences (licence number 30/2621). As part of this process the work has undergone scrutiny and approval by the ethics committee at the Institute for Animal Health.

### Cell preparation, flow cytometry and sortin

Single-cell suspensions of lymphocytes were prepared from spleen, blood and tumor tissues by Histopaque-1083 (Sigma-Aldrich, Steinheim, Germany) density-gradient centrifugation. CD4+ and CD8+ T cell populations were isolated by positive magnetic cell sorting (AutoMACS Pro Separator, Miltenyi Biotec, Bergisch Gladbach, Germany) according to manufacturer's instructions using FITC conjugated mouse anti-chicken CD4, clone CT-4 and anti-chicken CD8β antibodies, clone EP42 [[53]; SouthernBiotech, Birmingham, Alabama, USA)] and goat anti-mouse IgG microbeads (Miltenyi Biotec). After each antibody treatment, cells were washed three times with PBS containing 0.5% bovine serum albumin with centrifugation at 450 x*g* for 10 min. The purity of sorted cells was >99% by flow cytometry.

### Cell culture and maintenance of established cell lines

Established lymphoma cell lines derived from MDV-1-induced tumors included MSB1[54], HP8 [55] and HP18 [56], RPL-1 [57]. Four additional MDV cell lines were established from four line P birds infected with pRB-1B5 [51], from testes (T), ovary (O) and spleen (S) tumors according to standard methods [56]. These have been given the following identifiers 4523(T), 4525(O), 4590(S) and 760(O). The Reticuloendotheliosis virus T (REV-T strain)-transformed CD4+ T-cell line AVOL-1 [58], [59] was included as a MDV-negative transformed cell line. Cell lines were grown at 38.5°C in 5% CO_2_ in RPMI 1640 medium containing 10% fetal calf serum, 10% tryptose phosphate broth and 1% sodium pyruvate.

### RNA isolation

Tissue samples were stored in RNAlater (QIAGEN Ltd. Crawley, United Kingdom) at −20°C before disruption by homogenization (Mini-bead beater; Biospec Products, Bartlesville, Okla.). Isolated cell subsets or cultured cells were disrupted by resuspension in RLT buffer (QIAGEN) and stored at −20°C. RNA was extracted with the RNeasy Mini kit (QIAGEN) according to the manufacturer's instructions. Contaminating DNA was digested on column with RNase-free DNase 1 (QIAGEN) for 15 min at room temperature. The RNA was eluted with 50 µl RNase-free water (QIAGEN) and stored at −80°C.

### Reverse transcription

Reverse transcription reactions were performed using the iScript Reverse Transcription system (iScript Select cDNA synthesis Kit, Bio-Rad, USA) according to manufacturer's instructions, using 2 µg of isolated RNA from each sample and oligo(dT) primers. Twenty µl of cDNA was obtained for each sample and stored at -20°C.

### Polymerase chain reaction (PCR)

PCR were performed according to standard protocols. Briefly, cDNA (2 µl) was incubated with 200 µm dNTP, 1.5 mM MgCl_2_, 1x reaction buffer [50 mM KCl, 20 mM Tris–HCl (pH 8.4)], 2 units Platinum Taq DNA polymerase (Invitrogen), 1 µl of each primer at 10 µM working concentration, in a 50 µl final reaction volume. The forward primer used for Vβ1 and Vβ2 was 5′ACAGGTCGACCTGGGAGACTCTCTGA CTCTGAACTG-3′ and 5′-CACGGTCGACGATGAGAACGCTACCCTGAGATGC-3′ respectively with a common Cβ reverse primer 5′ACAGGTCGACGTACCAAA GCATCATCCCCATCACAA-3′ [60]. The TCRβ locus lies on chromosome 1 with Vβ and Cβ primer design based upon genomic sequence (version 82; http://www.ensembl.org/Gallus_gallus) as described previously (45). The use of primers that lie in conserved regions of the TCR segments minimises any bias associated with PCR amplification. Sequence analysis of samples derived from uninfected birds reveals a polyclonal population of amplified TCR CDR3 with no evidence of PCR bias (45 and our unpublished data).

PCR conditions were as follows, one cycle, 94°C for 2 min, followed by 35 cycles of 94°C for 30 s, 50°C for 40 s and 72°C for 1 min, followed by one cycle at 72°C for 10 min using a G-storm thermocycler (Gene Technologies, Essex, UK) or Eppendorf mastercycler (Eppendorf, Hamburg, Germany). The amplified products were analysed by electrophoresis through 1% agarose (Sigma-Aldrich Ltd, Poole, UK) gels in 1x Tris-borate-EDTA buffer at 50 mA for 1 hr, and products visualized by staining with ethidium bromide (Bio-Rad, Ltd) or GelRed nucleic acid stain (Biotium).

PCR products were purified using QIAquick PCR purification kit (Qiagen Ltd) according to manufacturer's instructions. DNA was eluted in 50 µl nuclease free water and stored at −20°C.

### Cloning and sequencing of PCR products

To determine the sequence of the expressed Vβ-chain, PCR products were cloned directly into the pCR4-TOPO vector (Invitrogen) and used to transform competent *E. coli*, TOP10 (Invitrogen) according to the manufacturer's instructions. After incubation on selective LB agar plates containing 100 µg/ml Ampicillin (Sigma), single bacterial colonies were picked and screened for insert of correct size by PCR followed by agarose gel electrophoresis. Positive colonies were processed using the Qiagen Miniprep kit (Qiagen Ltd) and subsequently sequenced with plasmid-specific (M13 Forward; 5′-GTAAAACGACGGCCAG-3′or M13 reverse; 5′-CAGGAAACAGCTATGAC-3′) or Cβ specific reverse primer (5′-TGTGGCCTTCTTCTTCTCTTG-3′). Alternatively, the plasmid insert amplified by PCR was purified using QIAquick PCR purification kit (Qiagen Ltd) according to manufacturer's instructions and sequenced directly using a nested Cβ specific reverse primer (above). Sequencing was carried out by capillary electrophoresis on the CEQ 8000 sequencer according to the manufacturer's instructions (Beckman Coulter, Fullerton, CA).

Up to 22 (usually ∼15) independent sequences were obtained with each sample. The sample size (n) was chosen with reference to the coefficient of variation of the binomial distribution, which is proportional to 1/√n. This means that the increased precision obtained by raising sample size above ∼n = 15 rapidly reaches a point of diminishing return. Appropriate confidence limits for the repeated sequence frequencies were calculated using the Adjusted-Wald method for binomial proportinos [61]. All sequence data was considered with reference to data generated by spectratype analysis of the CDR3 length profile generated from the total population of cells examined.

### Spectratyping

To determine the CDR3 lengths of the amplified PCR products by spectratype analysis, a run-off reaction was performed as follows. Five µl of purified PCR product was incubated with 200 µm dNTP, 1 mM MgCl_2_, 1x reaction buffer [50 mM KCl, 20 mM Tris–HCl (pH 8.4)], 0.5 units Taq DNA polymerase (Invitrogen), 1 µl of a WellRED dye D4 (Sigma) labelled nested Cβ specific reverse primer (5′-TCA TCT GTC CCC ACT CCT TC-3′) at 4 µM working concentration in a 20 µl final reaction volume.

The reaction conditions were as follows, one cycle 95°C for 2 min, followed by 4 cycles of 57°C for 2 min and 72°C for 20 min using a G-storm thermocycler (Gene Technologies, Essex, UK) or Eppendorf mastercycler (Eppendorf, Hamburg, Germany). The run-off reaction products were diluted 5x with nuclease free water and 1 µl of the diluted product was mixed with 40 µl sample loading dye (Beckman Coulter, Fullerton, CA) containing 0.25 µl DNA size standard kit-600 (Beckman Coulter, Fullerton, CA). Samples were transferred into a 96 well plate, overlaid with mineral oil and immediately loaded into a capillary sequencer (CEQ8000 Genetic Analysis System, Beckman Coulter) for fragment analysis. For optimal results, samples were analysed using a modified fragment analysis program (Frag-4) by increasing separation time to 75 min. The data was compiled in CEQ8000 analysis module and for each sample the range of base pair lengths of products was identified and displayed as spectratype profiles. Peak size data was extracted from the fragment analysis software and transferred into Microsoft Excel. Chi-squared tests were used to test whether each CDR3 length distribution differed significantly from that obtained with uninfected birds (TCRVβ1 and TCRVβ2 from unsorted cells or those positively sorted for expression of CD4 or CD8β). The spectratype profiles derived from uninfected birds (n = 3 for each population) exhibited consistently broad CDR3 length distributions that were not statistically different to each other. Reference CDR3 length distributions were constructed for each population by calculating the mean proportion of signal obtained at each CDR3 length from uninfected samples.

## Results

### MD tumor cell lines are composed of monoclonal T cell populations

**Figure 1:**
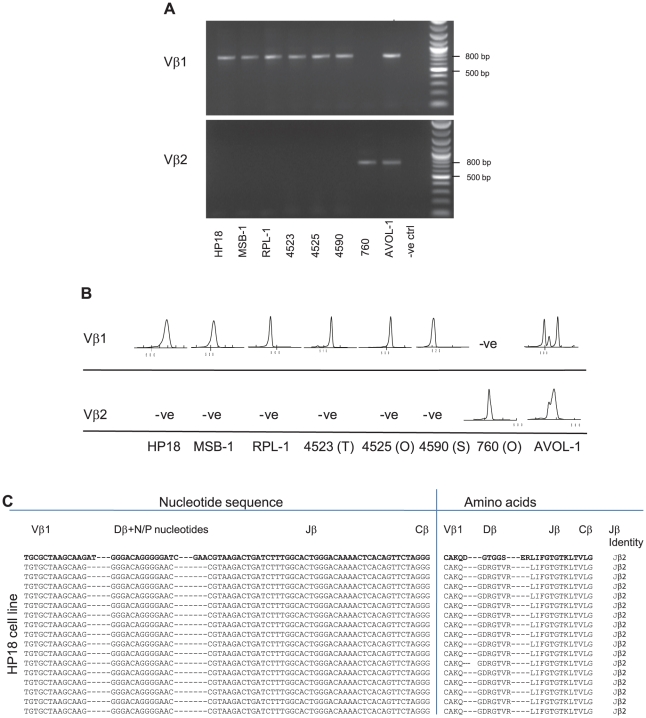
Clonality of established MDV cell lines revealed by TCRβ CDR3 repertoire analysis. RNA was prepared from seven MDV cell lines and one REV-transformed cell line (AVOL-1) and subjected to (A) RTPCR with products resolved on a 1% agarose gel and (B) spectratype analysis. Each sample was tested for expression of TCR Vβ1 and Vβ2 with specific primers. C) Sequencing 16 randomly picked clones of HP18 cell line confirms monoclonal status. The nucleotide sequences of the 3′ end of Vβ, whole Dβ (with N and P nucleotide modifications), whole Jβ and the 5′ end of Cβ (left column) and translated amino acid (aa) sequences (right column) are shown. For reference the top sequence (bold) is constructed in germ line configuration with Jβ2. All spectratypes were significantly different to the TCRVβ1 or TCRVβ2 reference profile for unsorted spleen cells obtained from uninfected birds (X^2^, p<0.001).

In the first instance we selected eight MDV-transformed cell lines [[54], [56], [57], [62] and our unpublished data] and subjected these to TCR repertoire analysis. The REV-T-transformed CD4+ T-cell line AVOL-1 [58], [59] was included for comparison. All of the MD tumor cell lines expressed either Vβ1 or Vβ2 exclusively, whereas the REV-T transformed AVOL-1 cell line expressed both TCR Vβ1 and Vβ2 (Figure 1A). The majority of the randomly selected cell lines (6/7) expressed Vβ1 suggesting a bias in tumor formation between the two avian TCRβ families. The spectratype-derived CDR3 length profiles for each MD cell line comprised a single spectral peak, whereas AVOL-1 contained multiple spectral peaks (Figure 1B). PCR products were cloned into the pCR4-TOPO vector and the inserts sequenced from single colonies of transformed *E. coli*. For each MD cell line, all inserts contained identical TCRβ CDR3 sequences whereas three sequences were obtained for Vβ1 in AVOL-1 (Figure 1C and S1). Taken together, these data indicate the clonal nature of MD cell lines compared with an oligoclonal structure in the REV-T transformed AVOL-1 cell line.

### Restricted clonality is evident in MD tumors

**Figure 2:**
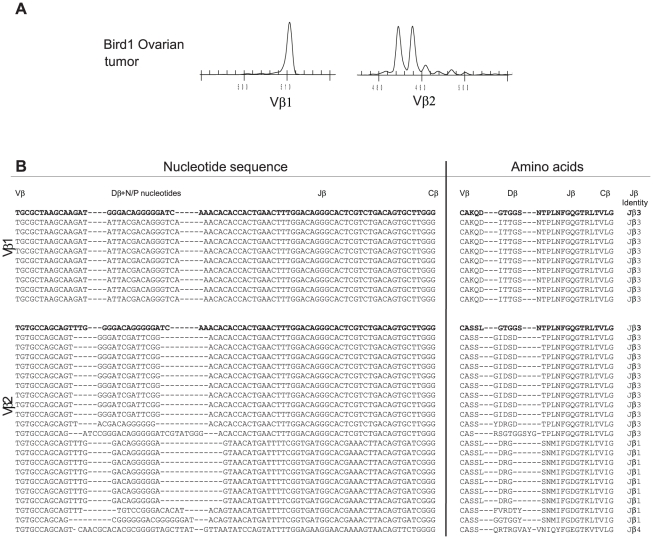
Restricted TCRβ repertoire in an ovarian tumor. Spectratype analysis on RNA isolated from an ovarian tumor from an MDV challenged Line P bird (90 dpi with pRB-1B), A) A single spectral peak for Vβ1 (left) and oligoclonal spectratype for Vβ2 (right) profile. B) Sequence analysis of Vβ1 CDR3 products (single clone) and an oligoclonal Vβ2 with two dominant CDR3 sequences at a frequency of 43% and 33%. The nucleotide sequences of the 3′ end of Vβ, whole Dβ (with N and P nucleotide modifications), whole Jβ and the 5′ end of Cβ (left column) and translated amino acid (aa) sequences (right column) are shown. The Jβ identity is indicated to right of aa sequence. For reference the top sequence (bold) is constructed in germ line configuration with Jβ3, the germ line aa sequences for Jβ are: Jβ1, SNMIFGDGTKLTVI; Jβ2, NVRLIFGTGTKLTVL; Jβ3, NTPLNFGQGTRLTVL; Jβ4, YVNIQYFGEGTKVTVL. All spectratypes were significantly different to the TCRVβ1 or TCRVβ2 reference profile for unsorted spleen cells obtained from uninfected birds (X^2^, p<0.001).

A fresh ovarian tumor was obtained from one pRB-1B5 MDV-infected bird (designated Bird1) at post mortem (90 DPI). Spectratype analysis revealed a restricted TCRβ repertoire (Figure 2A) with a single spectral peak for Vβ1. The Vβ2 spectratype profile of the ovarian tumor had two main peaks and 3 or 4 minor peaks. With Vβ1 all CDR3 sequences were identical (Figure 2B) corresponding in size to the CDR3 length observed by spectratyping, a profile similar to the tumor-derived cell lines. In contrast, with Vβ2 two repeated CDR3 sequences were detected, one which coded for the amino acid (aa) sequence ‘GIDSD’ at a frequency of 9/21sequences which translates to an estimate of 43% (_95%_CI 24-63%) of the population and the second, ‘DRG’ at 7/21 (33%, _95%_CI of 17–54% of the population). The remaining 5 sequences were singlets. The expanded Vβ2 clones may indicate presence of additional tumor clones, latently-infected T cells or a focussed T cell response infiltrating the tumor. These data demonstrate that MD tumor may consist of a monoclonal Vβ1 and an oligoclonal Vβ2 population. Application of spectratype and CDR3 sequence analysis to T cell populations from uninfected Line P birds revealed polyclonal repertoire profiles with no duplicated CDR3 sequence identified in any sample (data not shown).

### The dominant clones in MD tumors lie within culturable CD4+ cells

**Figure 3:**
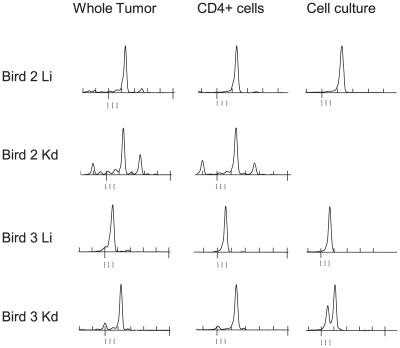
Dominant CDR3-lengths identified in tumors are present in sorted CD4+ cells and cultured cells. TCRVβ1 CDR3 length distribution (spectratype) within unsorted (left column) and CD4+ populations of cells derived from tumors (middle column) and cell lines established from three tumors (right column). Samples derived from liver and kidney of two Line P birds 32dpi with RB-1B MDV. The data indicates that the dominant spectral peaks in MDV tumors lie within a transformed population of CD4+ cells. All spectratypes were significantly different to the reference profiles for unsorted or CD4+ spleen cells obtained from uninfected birds (X^2^, p<0.001).

**Figure 4:**
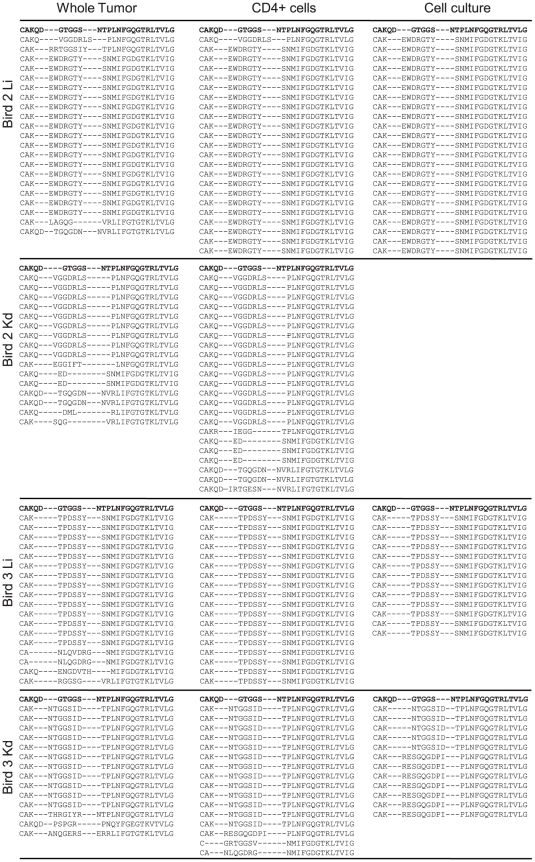
TCRVβ1 CDR3-sequence identity confirms clonal identity of T cells in tumor CD4+ and cultured cells. CDR3 amino acid sequences obtained by *in silico* translation of TCRVβ1 associated CDR3 nucleotide sequences. Samples derived from liver and kidney of two Line P birds 32 dpi with RB-1B MDV and represent unsorted tumors (left column) and CD4+ populations of cells derived from tumors (middle column) and cell lines established from three tumors (right column). Each sequence derives from cloned RTPCR product picked from single transformed colonies of *E. coli*. For reference the top sequence (bold) is constructed in germ line configuration with Jβ3, the germ line aa sequences for Jβ are: Jβ1, SNMIFGDGTKLTVI; Jβ2, NVRLIFGTGTKLTVL; Jβ3, NTPLNFGQGTRLTVL; Jβ4, YVNIQYFGEGTKVTVL. The data confirms clonal identity between MDV tumors and culturable CD4+ cells as suggested by spectratype analysis.

Since MDV transforms CD4+ cells [9]–[12], [14] we compared the CDR3 length distribution within unsorted and CD4+ populations of cells derived from tumors. Spectratype analysis of the liver and kidney tumors (32 DPI) from two additional individuals (designated Bird 2 and 3) revealed dramatic restriction in Vβ1 CDR3 length in unsorted cells (Figure 3, left column). These profiles were mirrored by the spectratypes of the CD4+ cell populations in all four tumor samples (Figure 3, middle column). Flow cytometry analysis showed that CD4 + cells represented between 88–98% of the cells derived from whole tumor (data not shown). Cell lines were established from three tumors, two of which had spectratype profiles identical to those detected within isolated CD4+ cells (Figure 3, right column). With the kidney tumor of Bird 3, the CDR3 spectra of cultured cells included a dominant peak of identical length to that in CD4+ T cells but also included a second slightly shorter peak. Sequence analysis revealed dominant sequences that were enriched by sorting for CD4+ cells and by *ex vivo* culture with the majority being derived from monoclonal expansions (Figure 4). The second spectral peak in the cultured cells of Bird 3 represented a second sequence detected once in the sorted CD4+ cells. Moreover, as a result of analysing two tumors from different organs from each individual, this data set also demonstrated that different tumor clones were present in different sites, with each site dominated by a single Vβ1 clone (e.g. CDR3 aa sequences EWDRGTY and VGGDRLS for Bird 2).

In contrast to Vβ1, the Vβ2 spectratype profiles of the 4 tumors (Figure S2) and corresponding sequences (Figure S3) indicate a wider repertoire although relatively large CD4 + T cell clones were detected in Bird 2 liver and kidney. However, none of these clones could be generated into transformed T-cell lines and may represent non-culturable tumors or a focussed T cell response. To identify the frequency of profiles consistent with metastatic tumor clones (shared clones in multiple sites) and those with independent origin (different clones), we carried out the spectratype analysis of multiple tumor sites from further seven birds (Bird 4 to 10). The profiles obtained for both Vβ1 and Vβ2 are shown in Figure S4 (A for Vβ1 and B for Vβ2). Dominant spectral peaks shared between multiple sites were found in 6 of 7 birds but there were also site-specific over-represented spectral peaks in most individuals, for example with the kidney Vβ1 of bird 7. Overall, the data indicate large bias in the profile of CDR3 length in all tumor sites (p<0.001) and the shared peaks between sites will often be due to a common CDR3 sequence. However, as seen with Bird 2 sometimes the sequence will be distinct despite shared CDR3 length (Figure 4). Interestingly, the dominant spectral peak seen in multiple tumor sites was often evident in spleen and/or blood samples supporting an interpretation of metastatic spread for some tumor clones.

Further spectratype and sequencing analyses were performed to identify the nature of the CD8+ response (see below), where cells from multiple tumor sites were sorted into CD4 and CD8 fractions. The spectratype profiles for whole tumor or sorted CD4+ cells from tumor sites in Birds 11 to 14 were similar to those seen with Bird 1 to 10, with dominant spectral peaks in tumor sites (Figure S5). Some of the dominant peaks were shared between tumor sites within a single bird whilst others were specific for particular sites. The Vβ1 and Vβ2 products were sequenced for all tumor sites in Birds 11 and 12 (Figures S6 to S9). In the absence of culturable T-cell lines generated from these tumors, we tentatively defined tumor-like clones as CD4-enriched and representing greater than 30% of the sequences in any one site (most were much higher frequency than 30%). Specifically, the sequence data for Vβ1 in CD4+ cells from Bird 11 (Figure S6) identifies three large tumor-like clones, “LDGTGGY” (liver only), “RRLTGD” (kidney and as a singlet in ovary) and “LDTGGS” (liver, kidney and ovary). The sequence for Vβ2 in CD4+ cells of Bird 11 (Figure S7) revealed one highly over-represented sequence in all sites (ILRDRGW) that may represent a metastatic tumor and a second in the spleen (IRLGTGGY). For Bird 12 (Figure S8) no Vβ1 CDR3 were represented at over 30% of CD4+ T cell derived sequences but one Vβ2 sequence (Figure S9) with the CDR3 motif “QG” was dominant in the kidney (18/19 CD4+ sequences) and detected in ovary and spleen. A second CD4+, Vβ2 CDR3 sequence “FVMRGID” was dominant in the ovary but not detected elsewhere.

In most individuals the sequencing approach revealed smaller clones of CD4+ cells (repeated but <30% of sequences in any site) including Vβ1 with Birds 2, 11 and 12 and in Vβ2 with Birds 3, 11 and 12 (Figures 4, S3, S6 to S). These sequences may also represent small tumor clones or responding cells but the expansion of one of these sequences in cultured cells from Bird 3 kidney indicates that the “small tumor clone” explanation is valid. Global attribution of the smaller clones of CD4 T cells to a response or tumor phenotype is not possible with the current data sets. Nonetheless, our data clearly demonstrated that culturable tumors were usually dominated by a single T cell clone but that different sites within the same individual can contain independent tumor clones.

### Large tumor clones can be identified in blood of MDV infected birds

**Figure 5:**
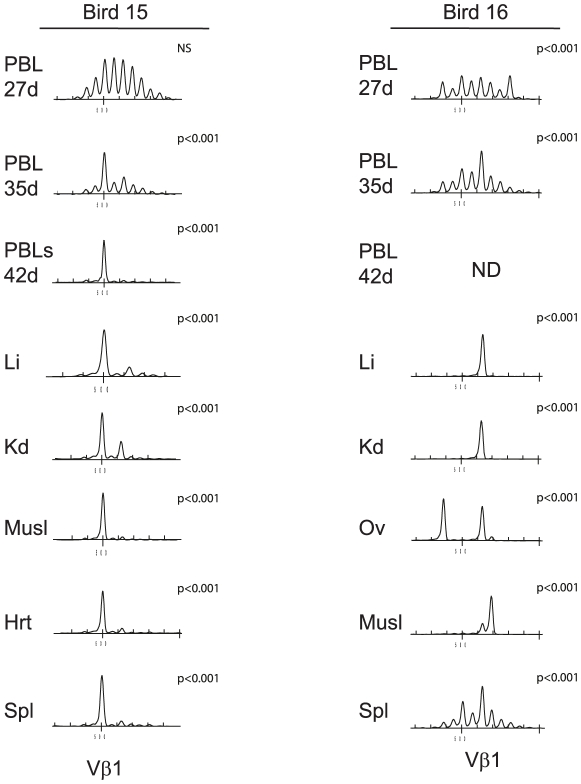
Spectratype analysis revealed that tumor clones can be identified in blood of MDV-infected birds. TCRVβ1 CDR3 length distribution of tumor, spleen and peripheral blood lymphocyte (PBL) samples from bird 15 (left column) and bird 16 (right column). Tumor and spleen samples were taken at post-mortem (49 dpi with RB1B MDV) and samples of PBL at 27 and 35dpi. Dominant spectral peaks could be detected in the blood at 35 DPI which correspond to the tumor profiles which contained the expected dominant spectral peaks. The spectratype distributions of samples were compared with the TCRVβ1 or TCRVβ2 reference profiles for unsorted or CD4+ spleen cells obtained from uninfected birds by X^2^ analysis; statistical significance is indicated with each panel. NS = not significant (at p>0.05). Li, liver; Kd, Kidney; Ov, ovary; Spl, spleen; Musl, muscle; Hrt, heart; PBL, peripheral blood lymphocytes.

The detection of tumor clones in the blood, at post-mortem raised the possibility of identifying tumor clones prior to the occurrence of overt disease. Initial analysis with samples of blood collected ∼2 weeks before the birds exhibited clinical signs supported the notion that the TCR spectratype would be useful to detect tumor clones circulating in the blood. The results of Vβ1 analysis of peripheral blood leukocyte (PBL) samples for two birds (Bird 15 and 16) are given in Figure 5. The samples from liver, kidney, muscle, heart and spleen taken at 49 DPI from Bird 15 revealed a dominant spectral peak that could also be detected in the blood at 42 and 35 DPI (leading to a significant bias in the spectral profile; p<0.001). Similarly, Bird 16 shared the same spectral peak in liver, kidney and ovary with an overrepresented peak and a biased CDR3 profile in the blood at 35 DPI (p<0.001), one week prior to the onset of clinical disease. In Bird 16, there was also a second spectral peak in the ovary and a non-shared spectral peak in the muscle that were not detected in the blood.

**Figure 6:**
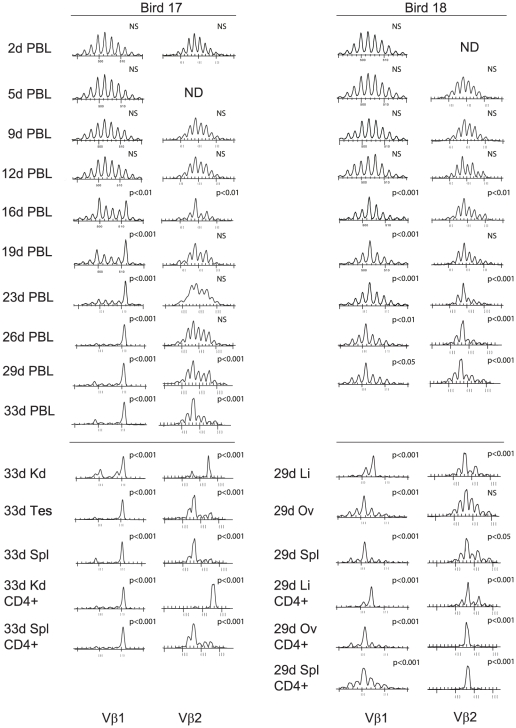
Early appearance of metastatic tumor clones in the blood of MDV-infected birds. TCRVβ1 and TCRVβ2, CDR3 length distribution of samples obtained from B17 (left columns) and B18 (right columns). Peripheral blood lymphocytes (PBL) were isolated from samples taken throughout infection with tumor, spleen and PBL samples taken at post-mortem. Some tumor and spleen samples were subjected to positive enrichment of CD4+ T cells by magnetic bead sorting. The dominant tumor associated spectral peaks could be detected in the PBL at the early stages of infection (e.g. bird 17 Vβ1 at 16 DPI). The spectratype distributions of samples were compared with the TCRVβ1 or TCRVβ2 reference profiles for unsorted or CD4+ spleen cells obtained from uninfected birds by X^2^ analysis; statistical significance is indicated with each panel. NS = not significant (at p>0.05). Li, liver; Kd, Kidney, Tes, testes, Ov, ovary; Spl, spleen; PBL, peripheral blood lymphocytes.

A further two birds (17 and 18) were blood sampled serially (twice a week) throughout infection for more precise detection of the tumor clones in the blood, and the results for Vβ1 and Vβ2 spectratypes are depicted in Figure 6. The tumor profile for Bird 17 at post-mortem (33 DPI) indicated a shared spectral profile for Vβ1 in kidney, testes and spleen (and in CD4+ cells isolated from kidney and spleen) and a second site-restricted tumor in the kidney comprising CD4+ Vβ2+ cells. The multi-site tumor CDR3 spectral length was readily detected in the PBL from 16 DPI (p<0.01 and at later time points p<0.001) whereas earlier PBL samples exhibited a “normal” distribution of CDR3 lengths that were not significantly different to the spectral profiles obtained from uninfected birds. In contrast, the site specific Vβ2 tumor was not detected as a spectratype bias in the PBL at any time. The tumor profiles of Bird 18 revealed one shared site (ovary and spleen) Vβ1 tumor, one single site Vβ1 tumor (liver) and one shared site Vβ2 tumor in all three sites (although the more complex ovarian tumor spectratype suggest it may be less highly represented). The multi-site Vβ1 tumor was detected as spectral bias in the PBL between 16 and 19 DPI (p<0.001) although the overall bias was less dramatic than seen with Bird 17.

The spectral profiles of PBL from MDV infected birds indicate that multi site tumor clones can be readily detected in the blood over two weeks prior to clinical symptoms. Unlike the multi site tumors, those restricted to a single site were not detected in the blood. The appearance of tumor clones in the blood affected the repertoire of the overall PBL population especially within the TCRVβ family that comprise the tumor (e.g. for Bird 17, the blood Vβ1 profile was completely dominated by the tumor). Moreover, the disturbance caused by a large CD4+ T cell tumor clone in Vβ1 also affected the repertoire profile of Vβ2 (compare pre- and post- 12 DPI spectratype profiles) with significantly altered CDR3-length profiles in the PBL of Bird 17 at 16 DPI (p<0.005), 29 DPI and 33 DPI (both p<0.001).

### MD tumors contain populations of highly focussed CD8+ cells

Although the nature of the tumor complicates identification of CD4+ T cell responses the CD8+ TCRαβ+ T cells clearly represent a responding T cell population capable of specific recognition, cytokine production and anti-MDV capability [38], [39], [63]. Moreover, in humans infected with persistent viruses (e.g. EBV, CMV and HTLV) the responding CD8+ T cells develop a highly focussed repertoire [2], [41]–[43], [64], [65]. Hence, to define the repertoire of the CD8+ response in MDV infected birds, we isolated CD8+ T cell populations from a range of tumor sites and subjected them to spectratype and sequence based repertoire analysis (simultaneous analysis of CD4+ populations was used to determine the nature of the tumor profiles in these individuals, Figures S5, S6, S7, S8, and S9).

**Figure 7:**
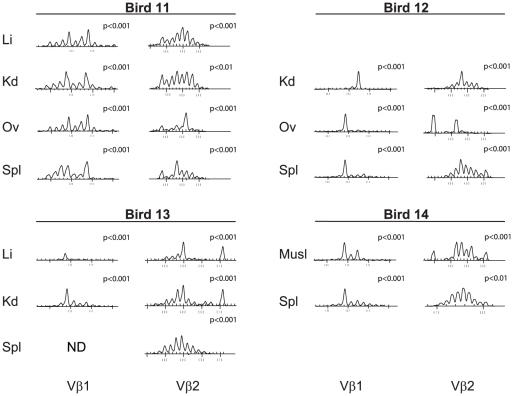
The responding CD8+ T cell repertoire is highly focussed in tumor sites and spleen. Spectratypes of TCR Vβ1 and Vβ2 transcripts from magnetically sorted CD8+ cells derived from multiple tumor sites of four birds (birds 11–14) are presented. The profiles show skewed distributions evident in all tumors sites within both Vβ1 and Vβ2, with some being shared between different tumors within a single host, some of which are also seen in spleen. These birds were also examined for CD4+ tumor clonality by spectratype (Figure S6) and CDR3 sequence (Figure S7) which revealed a combination of shared and tumor site-specific clones. The spectratype distributions of samples were compared with the TCRVβ1 or TCRVβ2 reference profiles from CD8β+ spleen cells obtained from uninfected birds by X^2^ analysis; statistical significance is indicated with each panel. NS = not significant (at p>0.05). Li, liver; Kd, Kidney; Ov, ovary; Spl, spleen.

**Figure 8:**
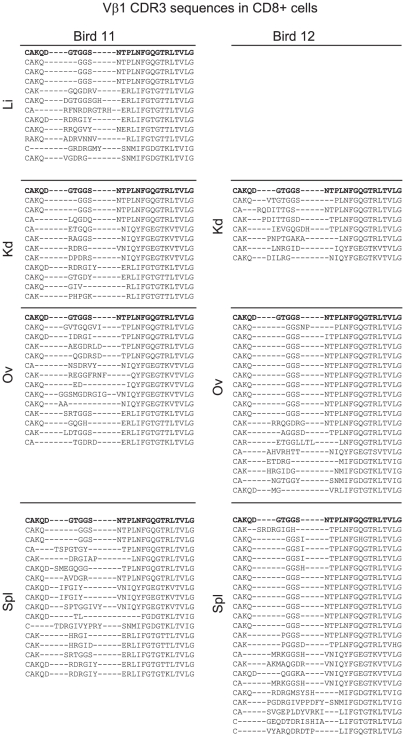
Private and public CDR3-sequences in CD8+ T cells derived from multiple tumor sites and spleen. CDR3 amino acid sequences obtained by *in silico* translation of TCRβ CDR3 nucleotide sequences for TCRVβ1 and: TCRVβ2. Samples derived from bird 11 and bird 12, magnetically sorted CD8+ cells (>99% pure) from multiple tumor sites and spleen. Each sequence derives from cloned RTPCR product picked from single transformed colonies of *E. coli*. Alignments are made with TCRVβ and Jβ regions, where possible remnants of the Dβ sequence are also used for alignment. For simplicity the top sequence (bold) is derived from translation of the genomic conformation of Vβ, Dβ and Jβ3. Translation of genomic sequences of Jβ segments are as follows: Jβ1, SNMIFGDGTKLTVI; Jβ2, NVRLIFGTGTKLTVL; Jβ3, NTPLNFGQGTRLTVL; Jβ4, YVNIQYFGEGTKVTVL.: The data reveals clonal structure within CD8+ T cell populations with shared clones between sites and a public TCRVβ1 CDR3 sequence shared between individuals.

Spectratype profiles obtained for Vβ repertoire analysis of CD8+ cells isolated from multiple tumor sites in four birds (11 to 14) are presented in Figure 7. CD8+ cells represented a minority cell population within the tumor, ranging between 0.4 and 5% by flow cytometric analysis (data not shown). Highly purified CD8+ cells (>99%) exhibited a restricted Vβ1 CDR3 length spectral profile (p<0.001; Figure 7). Within birds, the spectral profiles taken from different sites often included shared peaks detected in multiple samples. The Vβ2 spectral profiles were more variable but were also characteristic of biased populations (p<0.01 to p<0.001) with large over-represented peaks in some samples. The Vβ1 products were sub-cloned and sequenced from all sites in two birds (11 and 12) (Figure 8) allowing identification of clonal expansion by the presence of repeated sequences. These sequences included the CDR3 aa motif “GGS” present in both Bird 11 and 12 as a large, multi-site, overrepresented “public” CDR3 sequence. Considering this clone was the only sequence at this length in either Bird 11 or 12 it is intriguing that this spectral peak was also over-represented in the CD8+ T cells from Bird 13 and 14. Other repeated CDR3 sequences in CD8+ T cells included “RDRGIY” (in liver kidney and spleen), “SRTGGS” (ovary and spleen) and “IFGIY” (spleen) of Bird 11 and “GGSI” in the spleen of Bird 12. Further candidate CD8+ CDR3 sequences were identified as present in unsorted populations and not present in CD4+ sorted populations. These included those revealed by the Vβ2 sequencing efforts; two from Bird 2 (ETGGVY and FAFIDRGI), one from Bird 3 (TIERVD), two from Bird 11 (EVGEILY and TTPQGDRSQ) and one from Bird 12 (RGGYQPA).

Collectively, these results indicate a highly focussed CD8+ T cell response with some clones present at high frequencies in multiple tumor sites and the spleen. The tumor profile of Bird 11 (5 tumor-like clones, with two metastatic) and Bird 12 (2 tumor-like clones with one metastatic and one ovary-restricted) may relate to the identity of the CD8+ T cell expansions seen in different sites. For example the public GSS CDR3 sequence was detected at most tumor sites, whereas some other CD8+ clones were more restricted in their distribution to certain locations.

Based upon an assumption of similar TCR mRNA levels in all cells and the known numbers of Vβ1+ and Vβ2+ CD8+ cells in the tumor and spleen we can estimate the size of the CD8+ clones in the tumor site and spleen. For example, within Bird 11, the splenic population of the three CD8+ Vβ1+ clones “GGS” “RDRGIY” and “IFGIY” each represented 12.5% of the CDR3 which translate into populations of ∼25 million cells (_95%_CI 4–74×10^6^).. In Bird 12, the public CDR3 aa sequence “GGS” was present in 6/22 (27%) CDR3 sequences from CD8+ T cells representing ∼54 million cells (_95%_CI 24–96×10^6^) and the private “GGSI” represented in ∼27.2 million cells (_95%_CI 8–72×10^6^).

### Summary of CDR3 identified in MDV-infected birds or MDV transformed cell lines

**Figure 9:**
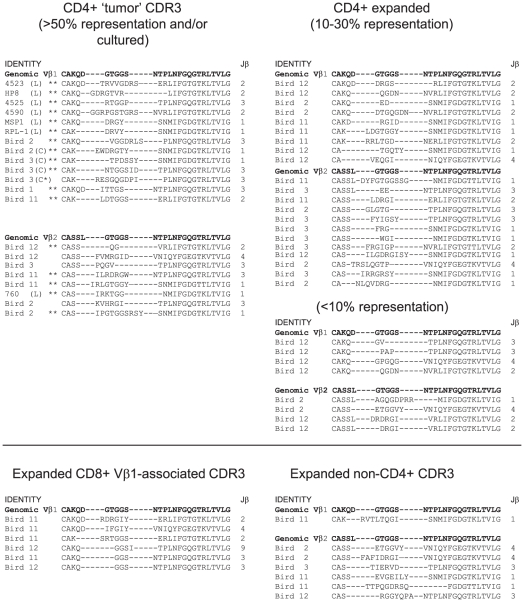
Summary of CDR3 identified in MDV-infected birds or MDV transformed cell lines. The CDR3 sequences are grouped according to Vβ usage and frequency detected in samples and/or the ability to grow in vitro (L =  established cell line; C = new cell line; C* =  new cell line from a small *in vivo* clone). ** CDR3 sequences detected at frequencies >70% of the sampled sequences. All data extracted from sequences presented in Figures 1, 2, 4, 7, S1, S3, and S6–S9. For comparison germ-line configuration of Vβ—Dβ—Jβ3Cβ sequence is given. Translation of genomic sequences of Jβ segments are as follows: Jβ1, SNMIFGDGTKLTVI; Jβ2, NVRLIFGTGTKLTVL; Jβ3, NTPLNFGQGTRLTVL; Jβ4, YVNIQYFGEGTKVTVL.: The identity of each Jβ is indicated to the right of each sequence.

For comparative purposes we have displayed the aa identity of all over-represented CDR3 sequences identified in this study and grouped these according to frequency in different T cell subsets (Figure 9). All cell lines contained monoclonal CDR3 sequences except for one short-term cultured cell line, which was biclonal. Within CD4+ T cells derived from tumor sites, fourteen high frequency CDR3 (>50%) were identified with ten represented at greater than 70% of the sequences obtained. Of the 21 “high frequency” CDR3 (established cell lines, ex vivo cultured cells and tumor sites), these were distributed in Vβ1 and Vβ2 based CDR3 (13 and 8 respectively). All four Jβ segments were represented. Other CD4+ CDR3 were present at 10 to 30% with a small number of low frequency (<10%) repeated sequences. Within positively sorted CD8 or non-CD4 (presumably CD8+) populations some large clones were detected, most of which represented private CDR3 but one represented a public CDR3 sequence detected in multiple birds. Samples of T cells from uninfected birds were polyclonal (no repeated CDR3) and none of the CDR3 seen in MDV infected birds was detected (data not shown).

## Discussion

Virus driven transformation of lymphoid cells is a major clinical consequence of infection with persistent infections such as EBV and HTLV in humans. Progress in understanding these human diseases is hindered by the lack of suitable model systems. MDV represents a natural α-herpesvirus of galliform birds capable of inducing rapid onset of tumors in susceptible birds. Losses caused by this group of viruses also represent a substantial problem in their own right; without MDV vaccination the poultry industry would be unsustainable. Indeed the ability to vaccinate against MDV tumor formation has implications for control of medically relevant tumors [1], [6]. Within this framework, we addressed the issue of T cell clonality during infection and tumor formation, dissecting the tumor, spleen and blood to identify repertoire changes in the transformed CD4+ cells and the responding CD8+ cells. With MD almost all cell lines and in vivo tumors have been characterised as CD4+, [9]–[11], [13], [14]. In one study using the intraperitoneal infection route one of twelve cell lines was CD4- CD8α+ but this lacked expression of CD8β [12]. All of the CD8+ samples in this study were prepared using anti-CD8β to avoid isolation of non-classical CD8αα T cells. We chose to examine the Vβ profiles as a measure of clonality since this receptor is clonally expressed with a single in-frame sequence present in each clone of T cells due to the process of allelic exclusion that takes place during T cell development in the thymus [66]–[68].

Tumor clonality is a fundamental issue in MD pathogenesis. The infectious cycle involves transfer of the virus from the lungs to initiate a cytolytic infection in B cells. This is followed by spread and lytic cycling infection largely within CD4 TCRαβ T cell population, before development of latent infection and transfer of MDV into the feather follicle epithelium from where the infectious virus is shed [8]. All infected birds experience a persistent, latent infection, and susceptible birds develop tumors usually within 4 to 5 weeks. Herein resides the problem; if the transformation event is rare, how to explain the high penetrance and temporal reproducibility of the tumor phenotype, unless the “tumors” are induced as a result of polyclonal transformation. Previous studies have addressed this issue in relation to the pattern of MDV genomic integration within the host cell genome [18], [19] or by cell surface staining with CD30 as a tumor associated marker [10]. These two studies reached opposing conclusions, with the restricted MDV integration profiles used to propose clonal tumors (with metastasis), contrasted with the high expression of CD30 in both TCRαβ families within a single tumor being used to propose polyclonality. Our studies using TCRVβ repertoire analysis techniques [45] as a viral integration independent clonal “bar-code” to identify the repertoire of CD4+ TCRαβ+ T cells in tumor-derived cell lines and with *in vivo* derived tumor samples revealed a characteristic of clonal dominance within an oligoclonal framework of tumor-capable CD4+ T cells.

All of the established tumor-derived cell lines were monoclonal (each expressing a single TCRβ CDR3 sequence), although one short-term line developed during the course of these studies was biclonal at second passage. In contrast the REV-T-transformed AVOL-1 cell line was oligoclonal after over 37 passages expressing at least three TCRVβ1 and one Vβ2 TCR CDR3 sequences. The spectratype profiles obtained with all cell lines were diagnostic in terms of the clonality of the CDR3 as defined by sequence analysis. The clonal structure of the cell lines was not influenced by the length of time in culture which suggests that monoclonality is not an artefact of *in vitro* selection as a result of multiple passages. It is therefore likely that selection for dominant transformed clones had already occurred *in vivo* and is retained in MDV cell lines as suggested previously [19]. Furthermore, cell lines generated in this study from fresh tumors expressed a TCR identity shared with the source tumor *in vivo*. Where cell lines were established most (∼90%) expressed the Vβ1 family of T cell receptors with only one expressing Vβ2, a ratio consistent with the 84% bias previously reported [13].

The profile of most primary tumors was dominated by a single clone of transformed T cells, although biclonal dominance in individual tumor sites was not uncommon. However, sequence analysis revealed smaller secondary clones of expanded CD4+ T cells in most tumors (∼10% of the CDR3 sequences) and the outgrowth of one of these during *ex vivo* culture indicates the tumor potential of sub-dominant CD4+ clones. Some of the very large clonal populations also failed to establish as tumor cells lines *ex vivo*, perhaps indicating a phenotypic variability in transformation state. Indeed, considering the very large TCR clones (40 to 100% of CD4+ CDR3 in one site) these were evenly distributed between TCRVβ1 (9 sequences) and TCRVβ2 (8 sequences) (Figure 9). The bias in TCRVβ usage within cell lines may represent a cultivation artefact or reflect the biology of cells expressing different TCR family members. Nonetheless, the multi-step analysis of dominant CDR3 in the primary tumor, in sorted CD4+ cells and after establishment of lymphoblastoid cell lines *ex vivo* are important in confirming the capacity of the identified large clones to express a tumor capability. All four Jβ segments were present in the CDR3 of both TCRVβ1 and TCRVβ2 expressing large tumor-like clones or in cultured tumor cell lines.

Our data resolves many of the issues surrounding MD tumor clonality. Essentially, we demonstrate clonal dominance within MD tumors (broadly similar to that reported by Delacluse et al., [19]) although our integration-site independent analysis using the T cell receptor CDR3 region revealed a more complex clonal framework within, and between, tumor sites *in vivo*. Different tumor sites within a single individual may be dominated by shared or distinct clones, hence a single individual may experience multiple transformation events giving rise to tumors that have very different characteristics. On most occasions the dominant in vivo clone present at a particular site was the only clone represented in *ex vivo* cultured cell lines grown under tumor culture conditions. However, on one occasion one of the lower frequency clones exhibited tumor-like growth patterns ex vivo (alongside the dominant clone in the original site) indicating that some of the smaller clones exist in a transformation capable state. The fact that many individuals harbour both metastatic and single-site tumor clones indicates a complex interplay between transformation and clonal competition. Indeed, with most individuals the overall tumor burden was the result of a small number of independent transformation events (i.e. more than one but fewer than 3 or 4). In contrast, with some individuals the multi-site tumors were the result of metastasis from a single tumor clone. The relationship between these “successful” tumor clones and the infected cell population deserves attention.

In a broader context, the monoclonal origin of adult T-cell leukaemia/lymphoma (ATLL) induced by the human T-lymphotropic virus type -1 (HTLV-1) associated malignancy is well documented [2], [69], [70]. This profile is probably related to the rarity of ATLL even among HTLV-1 seropositive individuals [3] reflecting the acquisition of secondary genomic lesions in persistently infected T cells. Nonetheless, the rapid onset MD tumors with clonal dominance in the context of a more complex framework of oligoclonal expansion may also reflect a circumstance common to other tumor associated persistent viruses of lymphocytes including HTLV-1. Perhaps the main differences may lie in the vigor of MDV-induced T cell replication leading to a compressed time-frame compared with other lymphotropic, tumor associated viruses.

Biological differences were also detected amongst the very large clonal CD4+ “tumors”, with some clones found in multiple sites including the blood and spleen whereas others were located in a single site, indicating phenotypic diversity based upon metastatic capability. The identification of metastatic tumor clones in the blood allowed serial analysis of blood samples from infected birds to determine the dynamics of the appearance of the tumor clone, in relation to the time of infection and onset of clinical signs. The spectratype analysis of blood samples prior to infection and in the first 10–14 days revealed a profile consistent with a polyclonal population of circulating cells. However in some cases, the ‘tumor-specific’ spectratype signature could be detected in blood 12 to 16 dpi, more than two weeks before appearance of clinical signs. The appearance of the tumor clone at detectable levels in the blood supports the proposal of an early transformation event. The level of tumor clone expansion in the blood compartment at the onset of clinical disease was extreme, and in some individuals, these were the only T cell clones detectable (e.g. within TCRVβ1 for Bird15 and 17) represented the tumor (Figure 5 and 6). There was also evidence for disturbance within the polyclonal repertoire in TCRVβ2 expressing cells (Figure 6) suggesting that the blood niche for T cells was being filled by the tumor. Hence, with a circulating TCR profile dominated by a single clone, it is of little surprise that MDV-infected birds develop immune deficiency [reviewed in [71]]. These dramatic repertoire changes would have greater impact than the reported changes in cytokine production [72] and would be immunologically catastrophic. Infiltration of the skin with CD4+ T cells is a consequence of MDV infection [73], [74] [75] and the high frequency tumor clones in the blood are likely to represent the relocation of MDV to the site of transmission.

In mammals, many persistent viral infections including EBV, CMV and HTLV stimulate highly focussed repertoire expansion in responding CD8+ T cells [2], [76], [77]. Although the MDV tumors were populated by relatively small numbers of CD8+ T cells, their repertoire was highly structured and oligoclonal in nature. The CD8+ T cell clone sizes of around 25 to 50 million cells are similar to those reported during persistent viral infections in humans [78]. However in the case of MD, these are developed over a much shorter period of time than considered with mammalian infections. For example, taking a conservative estimate of prolonged T cell division of 12 hours/division [79] and assuming no cell death (unlikely), the latest time point for initial stimulation of the CD8+ T cell would be ∼15 days prior to sampling. This calculation would place the initiation of these clones of specific CD8+ T cells at ∼15 DPI, probably earlier, around the time at which latent infection was initiated. The rapid focussing and clonal expansion of the MDV-specific repertoire suggests restriction to a small selection of MDV antigens. Indeed, Omar and Schat [38] examined the cytolytic response of infected birds against a panel of cell lines expressing individual genes from MDV found that in MHC B^19^ homozygote Line P_2a_ birds, the cytolytic activity was restricted to meq, gB and pp38 antigens, while the genetically-resistant line N_2a_ (B^21^) birds also detected the ICP4 antigens. In our studies, tumor-infiltrating CD8+ T cells produce greater levels of IFNγ mRNA than CD8+ T cells derived from the spleen of uninfected birds (unpublished data, Mwangi, Peroval et al.,). The CD8+ T cell response of susceptible birds is insufficient to prevent tumor progression; our data provides a framework for comparisons with resistant or vaccinated birds which do not develop tumors. Our sequence analysis clearly detected large CD8+ T cell clones and allowed an approximation of the clone size, the application of higher throughput sequencing technologies may be useful in the future to identify smaller clonal expansions and provide more accurate estimations of clone sizes. Understanding the nature of the TCR repertoire to specific antigens after infection and vaccination can be used to improve vaccine approaches in the future. The rapid nature of focussing within the CD8+ population may reflect a combination of the minimal MHC configuration where each haplotype is dominated by presentation through a single MHC class I gene [80] and the minimal TCRVβ locus with 13 Vβ segments in just two families [45].

The high frequency CD8+ T cell clones were found in both tumor sites and in the spleen of infected individuals, either restricted to one tumor site or present in multiple tumor sites. One of the largest CD8+ clones has a CDR3 sequence (“GSS”) of note, in that identical sequences were detected in different individuals. This type of CDR3 is known as a “public” TCR rearrangement and, although previously reported with mammals, is relatively rare [81]. Upon closer examination, it was clear that the public GSS amino acid sequence for the CDR3 also represented shared nucleotide sequence in different individuals. Interestingly, the GSS sequence represents retention of a fragment of the D segment, after deletion of six nucleotides in the D and three nucleotides in the Vβ1 segment. Although not noted previously, it is clear that a CDR3 constructed by deletion (with no retained nucleotide addition) is much more likely to occur in multiple individuals than one generated by addition of nucleotides. We propose that public CDR3 sequences in other contexts (e.g. in humans) may also conform to this arrangement, representing a deletion-based junctional modification. This feature might be useful and exploitable in diverse scenarios to improve “public” responses to vaccines. The remaining CDR3 sequences positively identified as clonal expansions in CD8+ cells (or as not in CD4+ cells) all represented “private” CDR3 identities (Figure 9).

In this report, we have documented the TCR Vβ repertoire changes associated with infection, tumor development and anti-tumor response that characterise MDV pathogenesis. Upon consideration of our data in the context of previous reports, we propose that the MD tumors are dominated by clonal expansion in an oligoclonal framework of minor clones of pre-cancerous cells. We propose that this type of population structure explains the penetrance and narrow temporal window that characterise MD in susceptible birds. The CDR3 analysis identified that all established MDV-transformed cell lines tested were clonal (with one bi-clonal short term culture), and that these clones represent dominant clones detected *in vivo*. Within birds harbouring multiple tumors there was a mixture of metastatic and site-specific tumor clones. Overall, we examined 50 tumors derived from 21 individuals, and all tumors were dominated by one or two clones with some birds harbouring a single metastatic tumor clone and others with different clones in different sites. The TCR repertoire analysis system has allowed examination of diverse areas of MD lymphoma biology and the CD8+ response against the infection. We consider that this type of approach can be used to further define MD pathogenesis and the response generated against infection and/or tumors. These types of study also have the potential to impact much more broadly, identifying strategies to vaccinate against or otherwise control viral driven lymphomas in medical and veterinary fields.

## Supporting Information

Figure S1 Monoclonal CDR3 sequences in MDV cell lines. Amino acid sequence alignment of TCRβ CDR3 for each cell line obtained by *in silico* translation of TCRβ CDR3 nucleotide sequences derived from 13–15 independent cloned plasmid inserts. For reference the top sequence (bold) in each alignment represents an unmodified germline sequence with appropriate Jβ. B) The TCRβ CDR3 nucleotide sequences from each cell line. The top sequence in each alignment (bold) in Vβ^1^ and Vβ^2^ is a constructed germline sequence (Vβ-Dβ-Jβ-Cβ). The total number of repeats for each sequence as a fraction of all sequences and Jβ identity is indicated to the right of the sequence.
(1.63 MB EPS)

Figure S2 Complex TCRVβ^2^ CDR3-length profiles in tumors and sorted CD4+ cells from birds 2 and 3. TCRVβ^2^ CDR3 length distribution within samples analysed in Figure 3 (for TCRVβ^1^), unsorted (left column) and CD4+ populations of cells derived from tumors (middle column). No product was obtained from cultured cells. Samples derived from liver and kidney of two Line P birds 32 dpi with RB-1B MDV. Although the distribution of spectral peaks was biased from that observed in unsorted or CD4+ spleen cells from uninfected birds (X^2^, p<0.001) no TCRVβ^2^ signal was represented in the transformed, cultured cells.
(0.88 MB EPS)

Figure S3 Oligoclonal CDR3-sequence repertoire of TCRVβ^2^ in tumors and CD4+ cells from birds 2 and 3. CDR3 amino acid sequences obtained by *in silico* translation of TCRVβ^2^ associated CDR3 nucleotide sequences. Samples derived from liver and kidney of two Line P birds 32 dpi with RB-1B MDV and represent unsorted tumors (left column) and CD4+ populations of cells derived from tumors (middle column) and cell lines established from three tumors (right column). Each sequence derives from cloned RTPCR product picked from single transformed colonies of *E. coli*. Alignments are made with TCRVβ^2^ and Jβ regions, where possible remnants of the Dβ sequence are also used for alignment. For simplicity the top sequence (bold) is derived from translation of the genomic conformation of Vβ^2^, Dβ and Jβ^3^. Translation of genomic sequences of Jβ segments are as follows: Jβ^1^, SNMIFGDGTKLTVI; Jβ^2^, NVRLIFGTGTKLTVL; Jβ^3^, NTPLNFGQGTRLTVL; Jβ^4^, YVNIQYFGEGTKVTVL.: The data indicates an oligoclonal repertoire and identifies some TCRVβ^2^ CDR3 associated with CD4+ T cells and others not represented in CD4+ cells.
(1.98 MB EPS)

Figure S4 Dominant CDR3-lengths are detectable in tumors from a further seven birds. Spectratype based CDR3-length distribution of TCR Vβ_1_ (A) and Vβ_2_ (B) in liver, kidney, ovary, muscle tumors for seven birds (B4-10). Spleen and peripheral blood lymphocytes obtained at post mortem are included. Skewed distributions are evident in all tumor sites compared with Vβ_1_ and Vβ_2_ profiles from uninfected birds (X^2^, p<0.001). Some peaks are shared between different tumors within a single host, some of which are also seen in spleen and PBL. Li, liver; Kd, Kidney; Ov, ovary; Musl, muscle; Spl, spleen; PBL, peripheral blood lymphocytes.
(2.18 MB EPS)

Figure S5 The spectratype profiles of TCRVβ from eight tumor sites in four birds. TCRVβ^1^ (left panel) and TCRVβ^2^ (right panel) CDR3 length distribution within unsorted and CD4+ populations of cells derived from tumors and spleen (Birds11–14). Samples from these birds were used in analysis of CD8+ TCR repertoires (Figure 7). The data confirms the presence of dominant clones in CD4+ T cells derived from tumor sites (compared with profiles obtained from uninfected birds) and that some dominant peaks were shared between tumor sites (within a single individual) whilst others were site-specific. The spectratype distributions of samples were compared with the TCRVβ^1^ or TCRVβ^2^ reference profiles from CD8β+ spleen cells obtained from uninfected birds by X^2^ analysis; statistical significance is indicated with each panel. NS = not significant (at p>0.05). Li, liver; Kd, Kidney; Ov, ovary; Spl, spleen.
(2.37 MB EPS)

Figure S6 TCRVβ^1^ CDR3-sequence identity from tumor and spleen samples for bird 11. CDR3 amino acid sequences obtained by *in silico* translation of TCRβ CDR3 nucleotide sequences, unsorted tumors (left column) and CD4+ populations of cells (right column) derived from tumors and spleen. Each sequence derives from cloned RTPCR product picked from single transformed colonies of *E. coli*. Alignments are made with TCRVβ and Jβ regions, where possible remnants of the Dβ sequence are also used for alignment. For simplicity the top sequence (bold) is derived from translation of the genomic conformation of Vβ, Dβ and Jβ^3^. Translation of genomic sequences of Jβ segments are as follows: Jβ^1^, SNMIFGDGTKLTVI; Jβ^2^, NVRLIFGTGTKLTVL; Jβ^3^, NTPLNFGQGTRLTVL; Jβ^4^, YVNIQYFGEGTKVTVL.: The data confirms that the dominant clones in MDV tumors lie within CD4+ cells and that within a single bird some dominant clones are shared with more than one site while others were site-specific. Samples from bird 11 were also used in analysis of CD8+ TCR repertoires (Figure 7).
(1.74 MB EPS)

Figure S7 TCRVβ^2^ CDR3-sequence identity from tumor and spleen samples for bird 11. CDR3 amino acid sequences obtained by *in silico* translation of TCRβ CDR3 nucleotide sequences, unsorted tumors (left column) and CD4+ populations of cells (right column) derived from tumors and spleen. Each sequence derives from cloned RTPCR product picked from single transformed colonies of *E. coli*. Alignments are made with TCRVβ and Jβ regions, where possible remnants of the Dβ sequence are also used for alignment. For simplicity the top sequence (bold) is derived from translation of the genomic conformation of Vβ, Dβ and Jβ^3^. Translation of genomic sequences of Jβ segments are as follows: Jβ^1^, SNMIFGDGTKLTVI; Jβ^2^, NVRLIFGTGTKLTVL; Jβ^3^, NTPLNFGQGTRLTVL; Jβ^4^, YVNIQYFGEGTKVTVL.: The data confirms that the dominant clones in MDV tumors lie within CD4+ cells and that within a single bird some dominant clones are shared with more than one site while others were site-specific. Samples from bird 11 were also used in analysis of CD8+ TCR repertoires (Figure 7).
(1.64 MB EPS)

Figure S8 TCRVβ^1^ CDR3-sequence identity from tumor and spleen samples for bird 12.CDR3 amino acid sequences obtained by *in silico* translation of TCRβ CDR3 nucleotide sequences, unsorted tumors (left column) and CD4+ populations of cells (right column) derived from tumors and spleen. Each sequence derives from cloned RTPCR product picked from single transformed colonies of *E. coli*. Alignments are made with TCRVβ and Jβ regions, where possible remnants of the Dβ sequence are also used for alignment. For simplicity the top sequence (bold) is derived from translation of the genomic conformation of Vβ, Dβ and Jβ^3^. Translation of genomic sequences of Jβ segments are as follows: Jβ^1^, SNMIFGDGTKLTVI; Jβ^2^, NVRLIFGTGTKLTVL; Jβ^3^, NTPLNFGQGTRLTVL; Jβ^4^, YVNIQYFGEGTKVTVL.: The data confirms that the dominant clones in MDV tumors lie within CD4+ cells and that within a single bird some dominant clones are shared with more than one site while others were site-specific. Samples from bird 12 were also used in analysis of CD8+ TCR repertoires (Figure 7).
(1.64 MB EPS)

Figure S9 TCRVβ^2^ CDR3-sequence identity from tumor and spleen samples for bird 12. CDR3 amino acid sequences obtained by *in silico* translation of TCRβ CDR3 nucleotide sequences, unsorted tumors (left column) and CD4+ populations of cells (right column) derived from tumors and spleen. Each sequence derives from cloned RTPCR product picked from single transformed colonies of *E. coli*. Alignments are made with TCRVβ and Jβ regions, where possible remnants of the Dβ sequence are also used for alignment. For simplicity the top sequence (bold) is derived from translation of the genomic conformation of Vβ, Dβ and Jβ^3^. Translation of genomic sequences of Jβ segments are as follows: Jβ^1^, SNMIFGDGTKLTVI; Jβ^2^, NVRLIFGTGTKLTVL; Jβ^3^, NTPLNFGQGTRLTVL; Jβ^4^, YVNIQYFGEGTKVTVL.: The data confirms that the dominant clones in MDV tumors lie within CD4+ cells and that within a single bird some dominant clones are shared with more than one site while others were site-specific. Samples from bird 12 were also used in analysis of CD8+ TCR repertoires (Figure 7).
(1.70 MB EPS)
